# Sex-related differences and associated transcriptional signatures in the brain ventricular system and cerebrospinal fluid development in full-term neonates

**DOI:** 10.1186/s13293-025-00719-2

**Published:** 2025-05-25

**Authors:** Yuxin Sun, Chenxin Fu, Lifan Gu, Huifang Zhao, Yuying Feng, Chao Jin

**Affiliations:** 1 Department of Radiology, The First Affiliated Hospital of Xi’an Jiaotong University, Xi’an, P. R. China; 2 Shaanxi Engineering Research Center of Computational Imaging and Medical Intelligence, Xi’an, P. R. China; 3 Xi’an Key Laboratory of Medical Computational Imaging, Xi’an, China

**Keywords:** Choroid plexus growth, Neonatal brain development, Ventricular system analysis, Right lateral ventricle, MRI analysis, LASSO regression, AHBA dataset, DERL2, MRPL48

## Abstract

**Background:**

The cerebrospinal fluid (CSF) is known to serve as a unique environment for neurodevelopment, with specific proteins secreted by epithelial cells of the choroid plexus (CP) playing crucial roles in cortical development and cell differentiation. Sex-related differences in the brain in early life have been widely identified, but few studies have investigated the neonatal CSF system and associated transcriptional signatures.

**Methods:**

This study included 75 full-term neonates [44 males and 31 females; gestational age (GA) = 37–42 weeks] without significant MRI abnormalities from the dHCP (developing Human Connectome Project) database. Deep-learning automated segmentation was used to measure various metrics of the brain ventricular system and CSF. Sex-related differences and relationships with postnatal age were analyzed by linear regression. Correlations between the CP and CSF space metrics were also examined. LASSO regression was further applied to identify the key genes contributing to the sex-related CSF system differences by using regional gene expression data from the Allen Human Brain Atlas.

**Results:**

Right lateral ventricles [2.42 ± 0.98 vs. 2.04 ± 0.45 cm3 (mean ± standard deviation), *p* = 0.036] and right CP (0.16 ± 0.07 vs. 0.13 ± 0.04 cm3, *p* = 0.024) were larger in males, with a stronger volume correlation (male/female correlation coefficients r: 0.798 vs. 0.649, *p* < 1 × 10^− 4^). No difference was found in total CSF volume, while peripheral CSF (male/female β: 1.218 vs. 1.064) and CP (male/female β: 0.008 vs. 0.005) exhibited relatively faster growth in males. Additionally, the volumes of the lateral ventricular system, third ventricle, peripheral CSF, and total CSF were significantly correlated with their corresponding CP volume (r: 0.362 to 0.799, *p* < 0.05). *DERL2* (Degradation in Endoplasmic Reticulum Protein 2) (*r* = 0.1319) and *MRPL48* (Mitochondrial Large Ribosomal Subunit Protein) (*r*=-0.0370) were identified as potential key genes associated with sex-related differences in CSF system.

**Conclusion:**

Male neonates present larger volumes and faster growth of the right lateral ventricle, likely linked to corresponding CP volume and growth pattern. The downregulation of *DERL2* and upregulation of *MRPL48* may contribute to these sex-related variations in the CSF system, suggesting a molecular basis for sex-specific brain development.

**Supplementary Information:**

The online version contains supplementary material available at 10.1186/s13293-025-00719-2.

## Introduction

Males and females differ in various ways, including physically, psychologically, and even in how diseases manifest and progress [1]. Understanding sex-related differences is crucial for developing tailored diagnostic approaches and therapeutic interventions, ultimately leading to more-personalized healthcare [2]. Sex-related differences in brain structure and neural processes may also contribute to the formation of masculine and feminine characteristics [3]. Advances in voxel-based morphometry have significantly expanded our knowledge of how sex influences the development, structure, and functioning of the brain [4]. One of the most consistent findings is that male brains are larger than female brains from birth, with this difference stabilizing at around 11% in adulthood according to multiple studies. However, whether specific brain structures also exhibit sex-related differences remains a topic of ongoing research [3].

The cerebral ventricular system comprises the lateral ventricles, third ventricle, and fourth ventricle and is continuous with the central canal of the spinal cord. CSF circulates within this system and the subarachnoid space [5]. The CSF is initially produced by the neuroepithelium during early development and later primarily by the CP, and has three primary functions: maintaining osmolarity, providing buoyancy, and facilitating waste clearance. Disruptions in CSF dynamics can not only reflect underlying brain changes but also exacerbate disease processes [6]. Alterations in the morphologies and sizes of the ventricles during neonatal development can indicate CSF space obstruction, reductions in brain parenchymal volume, or abnormal brain development due to issues with neuronal proliferation and migration [7]. Even minor ventricular enlargement has been associated with neurodevelopmental delays and neuropsychiatric disorders, which might not be detected until morphological changes or clinical symptoms become evident [8].

Knowledge of sex-related differences in the brain ventricular system and CSF development in neonates is essential for understanding brain development and improving early diagnostic approaches. A study of 98 fetuses found that the males had a larger left lateral ventricle [9]. However, another study involving 505 participants from birth to 18 years found that the brain-to-CSF volume ratio changed with age, with no significant sex-related differences regardless of whether or not body-size normalization was applied [10]. These inconsistencies, coupled with a focus on fetal development or extended age ranges, highlight the need for research specifically addressing neonatal CSF development, which has crucial clinical applications [11–13]. Furthermore, since the volumetric development of preterm neonates often deviates from normative values, it is important to investigate CSF development specifically in full-term neonates rather than grouping all healthy neonates together [14–16]. Research into the subdivision of the CSF system has been inadequate, largely due to challenges in sample acquisition and the accurate segmentation of fine structures. Additionally, studies have shown that GA impacts neonatal brain maturation and metrics of the brain ventricular system, necessitating consideration of the GA when examining the relationships between postnatal age and CSF volumes across sex group [11, 17].

The present study therefore examined the sex-specific relationships between postnatal age and specific CSF space metrics while accounting for the impact of GA, with the aim of further characterizing the underlying sex-related differences and associated transcriptional signatures in neonatal brain development.

## Method

### Participants

The dataset used in this study was obtained from the publicly available dHCP [18], and included MRI scans from 100 neonates (60 males and 40 females; GA = 28–42 weeks). All of the anatomical images were reviewed by a perinatal neuroradiology expert, who assigned a radiology score from 1 to 5 to each image, with 1 representing a normal appearance according to age, and 5 representing incidental findings with possible/likely significance for both clinical and image analyses. From this dataset, 75 full-term neonates (44 males and 31 females; GA = 37–42 weeks) without significant MRI abnormalities either for clinical or image analysis (radiology score ≤ 2) within 28 days after birth (postnatal age = 0–28 days) were selected. MRI scans were obtained using a Philips 3-T scanner at St. Thomas’ Hospital, London, UK with a 32-channel, neonate-dedicated head coil [19]. Details of the data-acquisition parameters are available at the dHCP website (http://www.developingconnectome.org/).

### Image processing

Neonatal T1-weighted images were processed using the uAI research portal(uRP). The uRP’s segmentation module offers notable advantages over conventional methods in speed (0.7 s per structure vs. 20 s), accuracy (average Dice coefficient: 96.6% vs. 84.3%), and robustness (success rate: 98.6% vs. 83.3%) [20]. The platform has already demonstrated promising results in neonatal brain segmentation, with several publications supporting its efficacy [21, 22]. The structural MRI data were automatically segmented using a 3D deep-learning model integrated in the platform [23, 24]. Sample segmentation results are shown in Supplementary Fig. 1.

The processing pipeline included (a) bias-field correction, (b) resampling to algorithmic space, (c) standardization of gray values to the range from − 1 to 1, (d) generation of a skull-stripped mask, (e) segmentation of white matter, gray matter, and CSF, (f) bilateral segmentation, and (g) parcellation into 109 subregions. The volumes of various brain regions, including of the total CSF, peripheral CSF, left and right lateral ventricles, left and right CP, third and fourth ventricles, and the total brain volume (TBV), were calculated using 3D Slicer software (version 5.6.1) [25].

The Evans Index (EI) was computed from the neonatal T1-weighted images as the ratio of the maximum transverse diameter of the frontal horns of the lateral ventricles to the largest internal diameter of the skull [26].

### Statistical analyses

Statistical analyses were conducted using SPSS (version 18). To assess the mean differences between males and females, we first tested the data for normality using the Shapiro-Wilk test. If *p* > 0.05, we assumed the data followed a normal distribution and proceeded with tests for homogeneity of variance. If any group deviated from normality, we used the Mann-Whitney U test. If the data were normally distributed with equal variances, we applied a two-sample t-test; if the variances were unequal, we used Welch’s t-test. The specific statistical methods applied to each group are detailed in Supplementary Table (1) We also test the differences between groups after adjusting for postnatal age and gestational age using a multiple linear regression test. For the analysis of CSF parameters across age, we first conducted linear correlation analysis between each CSF metric and GA, stratified by gender, shown in Supplementary Table (2) The residuals from these models represent the volumes adjusted for GA. We then performed correlation analysis on these adjusted volumes. If both male and female groups met the normality assumption (using the tests mentioned above), we applied Pearson’s correlation; otherwise, we used Spearman’s rank correlation. The same methodology was used to assess the correlation between choroid plexus (CP) volume and ventricular volumes. Linear regression models adjusted for GA were used to assess the relationships between ventricular metrics and postnatal age. We reported the R-squared values, regression coefficients (β), and *p* for β of the linear regression model. The regression lines were drawn as solid if *p* < 0.05 and as dashed lines otherwise. A *p* value of < 0.05 was considered statistically significant.

### Estimation of regional gene expression and lasso regression analysis

**Fig. 1:**
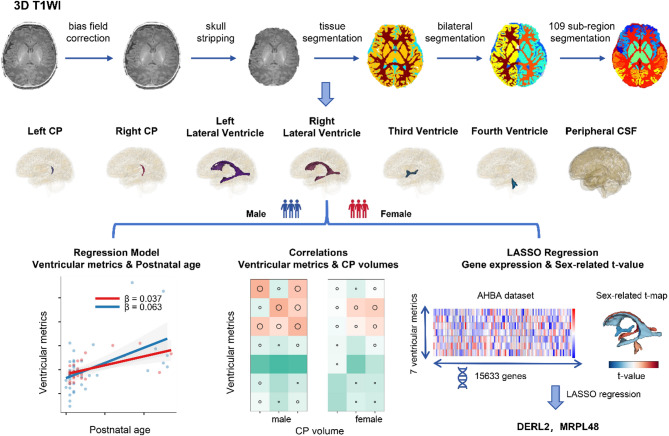
Study overview. T1-weighted images MRI scans from 75 full-term neonates were obtained from dHCP. Deep-learning automated segmentation was used to measure various metrics of the brain ventricular system and CSF. Sex-related differences and relationships with postnatal age were analyzed by linear regression. Correlations between the CP and CSF space metrics were also examined. LASSO regression was further applied to identify the key genes contributing to the sex-related CSF system differences by using regional gene expression data from the Allen Human Brain Atlas

Gene expression data from the Allen Human Brain Atlas (http://human.brain-map.org), a comprehensive whole-brain transcriptomic dataset, were processed using the abagen [27] toolbox to generate a regional expression matrix. The corresponding variables were the sex-related t-values for the volume of each ventricular region (calculated from a multiple regression model including GA, postnatal age, and sex). Least Absolute Shrinkage and Selection Operator (LASSO) regression was applied to construct predictive models. LASSO incorporates a penalty function that drives the coefficients of relatively unimportant variables toward zero, ultimately excluding them from the model. The penalty parameter (λ) was determined through five-fold cross-validation, with model selection based on the λ value corresponding to the lowest partial likelihood deviance (Fig. 1).

## Results

### Participant demographics

The demographics of the 75 full-term neonates without significant MRI abnormalities are presented in Table 1.

**Table 1:** Participant demographics

Clinical characteristics	Total	Male	Female	*p* value
Number	75	44	31	
Gestational age (w)	40.01 ± 1.07	39.98 ± 1.10	40.04 ± 1.03	0.820
Postmenstrual age (w)	40.77 ± 1.55	40.78 ± 1.61	40.76 ± 1.48	0.959
Postnatal age (d)	5.35 ± 7.56	5.57 ± 8.07	5.03 ± 6.89	0.507
Birth weight (kg)	3.37 ± 0.49	3.46 ± 0.49	3.24 ± 0.45	0.050

Full-term neonates with no significant abnormalities on MRI performed within 28 days after birth. Data are mean ± standard-deviation values

### Sex-related differences in the brain ventricular system and CSF space metrics

Males exhibited larger average values for several of the analyzed metrics, including the EI, right lateral ventricle volume, right CP volume (*p* < 0.05). However, no significant sex-related differences were observed in total CSF volume, which may be largely attributed to compensation from peripheral CSF space. Specifically, the percentages of the peripheral CSF and total CSF relative to TBV were 20.75% and 22.25% in females, respectively, and 20.05% and 21.64% in males (Table 2).

**Table 2:** Sex-related differences in metrics of the brain ventricular system and cerebrospinal fluid (CSF)

Brain ventricular system and CSF metrics	Total	Male	Female	*p* value
EI	0.25 ± 0.02	0.25 ± 0.02	0.24 ± 0.02	0.050*
Left CP	0.13 ± 0.06	0.14 ± 0.07	0.12 ± 0.05	0.429
Right CP	0.15 ± 0.06	0.16 ± 0.07	0.13 ± 0.04	0.024*
Total CP (cm^3^)	0.28 ± 0.10	0.30 ± 0.12	0.25 ± 0.07	0.075
Left Lateral Ventricle (cm^3^)	2.47 ± 0.77	2.56 ± 0.87	2.34 ± 0.6	0.249
Right Lateral Ventricle (cm^3^)	2.26 ± 0.82	2.42 ± 0.98	2.04 ± 0.45	0.036*
Total Lateral Ventricle (cm^3^)	4.73 ± 1.44	4.97 ± 1.66	4.38 ± 0.98	0.060
Third Ventricle (cm^3^)	0.29 ± 0.09	0.30 ± 0.10	0.28 ± 0.07	0.347
Fourth Ventricle (cm^3^)	0.41 ± 0.09	0.42 ± 0.10	0.39 ± 0.08	0.162
Peripheral CSF (cm^3^)	71.12 ± 13.91	71.91 ± 14.57	70 ± 13.08	0.559
Total CSF (cm^3^)	76.55 ± 14.64	77.61 ± 15.39	75.05 ± 13.62	0.430
TBV (cm^3^)	349.26 ± 39.43	358.14 ± 39.51	336.65 ± 36.30	<0.001*
Left CP/TBV (%)	0.04 ± 0.02	0.04 ± 0.02	0.04 ± 0.01	0.820
Right CP/TBV (%)	0.04 ± 0.01	0.04 ± 0.02	0.04 ± 0.01	0.110
Total CP/TBV (%)	0.08 ± 0.03	0.08 ± 0.03	0.08 ± 0.02	0.297
Left Lateral Ventricle/TBV (%)	0.71 ± 0.21	0.72 ± 0.25	0.69 ± 0.14	0.723
Right Lateral Ventricle/TBV (%)	0.64 ± 0.21	0.67 ± 0.25	0.61 ± 0.12	0.199
Total Lateral Ventricle/TBV (%)	1.35 ± 0.37	1.39 ± 0.44	1.30 ± 0.23	0.343
Third Ventricle/TBV (%)	0.08 ± 0.02	0.08 ± 0.03	0.08 ± 0.02	0.985
Fourth Ventricle/TBV (%)	0.12 ± 0.03	0.12 ± 0.03	0.12 ± 0.02	0.733
Peripheral CSF/TBV (%)	20.34 ± 3.12	20.05 ± 3.23	20.75 ± 2.95	0.300
Total CSF/TBV (%)	21.89 ± 3.23	21.64 ± 3.39	22.25 ± 3.02	0.368

Data are mean ± standard-deviation values

*, *p* < 0.05

Postnatal age and GA were adjusted using a multiple linear rergression

### Sex-related differences in the development of the brain ventricular system and CSF

Significant correlations were found between CSF space metrics and postnatal age. Most *p* values decreased after adjusting for GA. Positive correlations with postnatal age were observed for the volumes of the left (r-male = 0.358, r-female = 0.374), right (r-male = 0.468, r-female = 0.463), and total lateral ventricles (r-male = 0.488, r-female = 0.457), left CP in males (*r* = 0.321), right CP in females (*r* = 0.468), and total CP (r-male = 0.334, r-female = 0.454), third ventricle (r-male = 0.621, r-female = 0.588), peripheral CSF (r-male = 0.604, r-female = 0.736), and total CSF (r-male = 0.619, r-female = 0.757). Moreover, the percentages of the third ventricle, peripheral CSF, and total CSF relative to TBV showed significant positive correlations with postnatal age. These results were adjusted for GA.

**Fig. 2:**
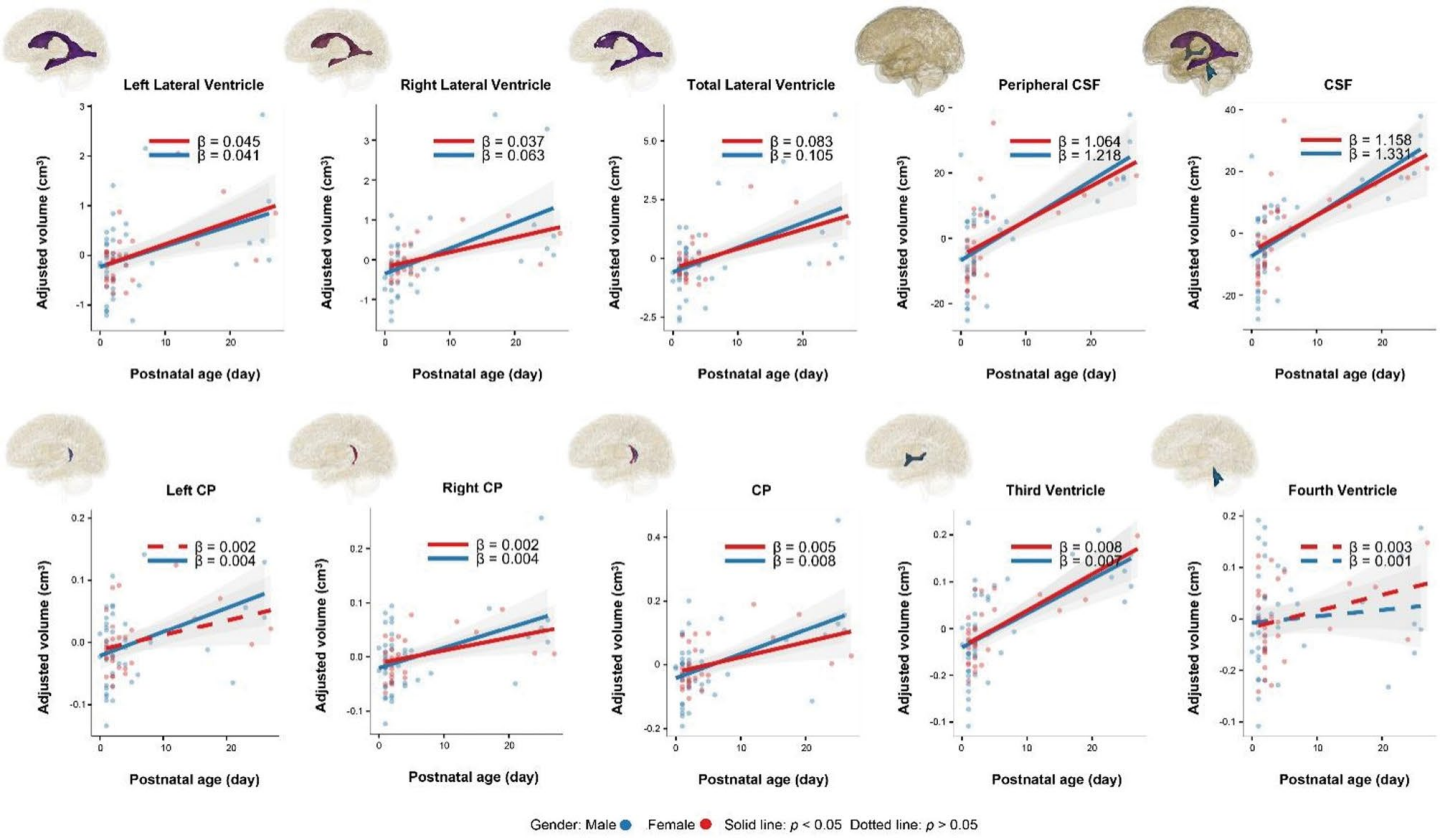
Scatterplots and linear regression curves illustrating the relationship between CSF volumes and postnatal age. These volumes were adjusted using residuals after performing linear regression with gestational age

**Fig. 3:**
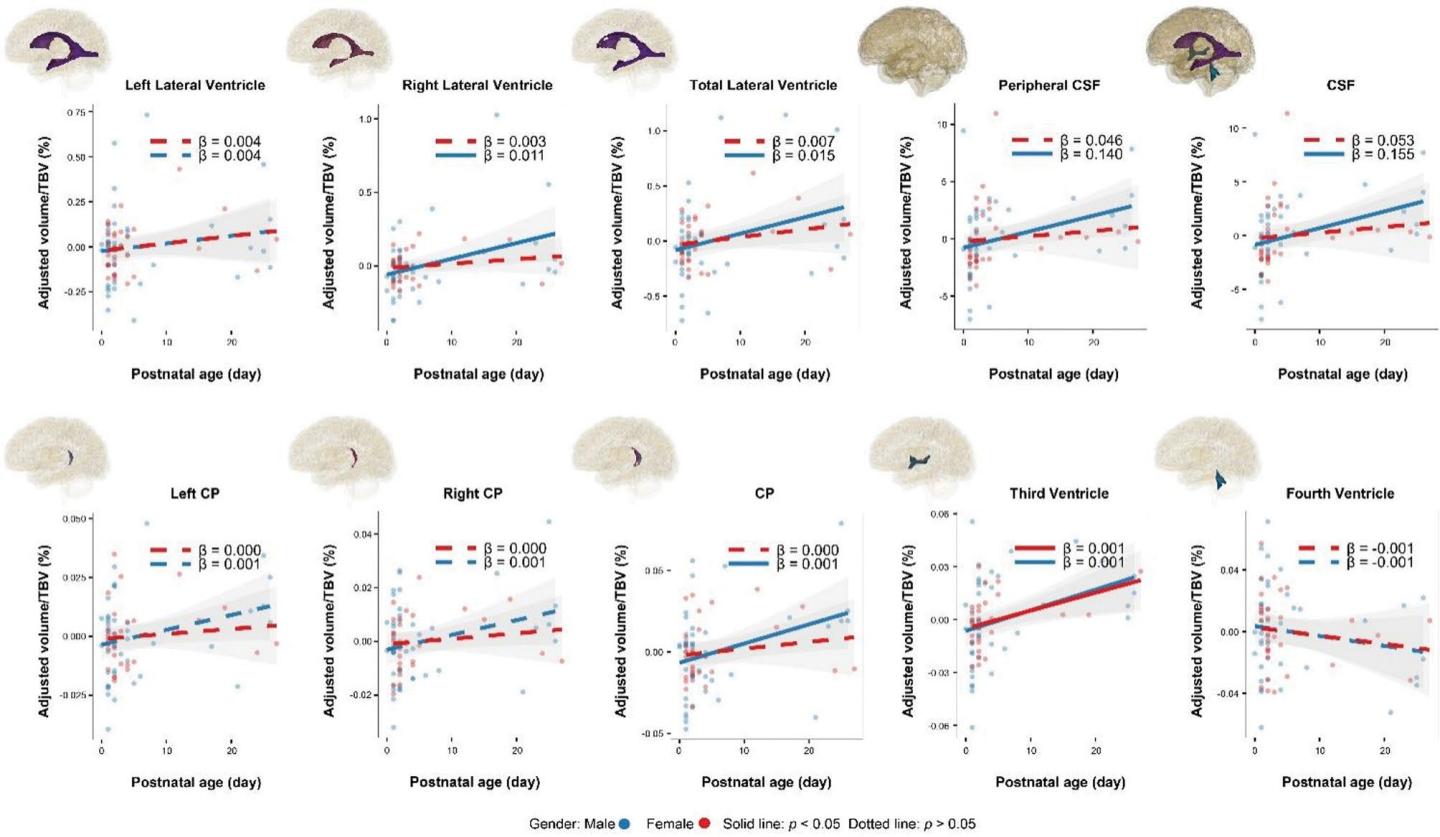
Scatterplots and linear regression curves illustrating the relationship between CSF volumes/TBV% and postnatal age. These percentages were adjusted using residuals after performing linear regression with gestational age

Males demonstrated faster growth rates of the right and total lateral ventricles than did females, with the right lateral ventricle showing the largest difference (regression coefficient β: males = 0.063, *p* < 0.001; females = 0.037, *p* < 0.001) (Fig. 2). Even when considering the volume of the right lateral ventricle as a percentage of the total TBV, the coefficients remained significant for males, with the *p* value for females exceeded the significance threshold of 0.05 (Fig. 3).

Similar patterns were observed for the peripheral and total CSF. The linear fit for the peripheral CSF as a percentage of TBV was not statistically significant in females, whereas males exhibited steady growth with β = 0.14 (Fig. 3).

Trends in the CP were consistent with those in total lateral ventricles, peripheral CSF, and total CSF across both their absolute volumes and percentages relative to TBV. Similar trends were also found between right CP and right lateral ventricles (Fig. 2).

### Correlations between CP volume and the brain ventricular system and CSF space metrics in different sex groups

**Fig. 4:**
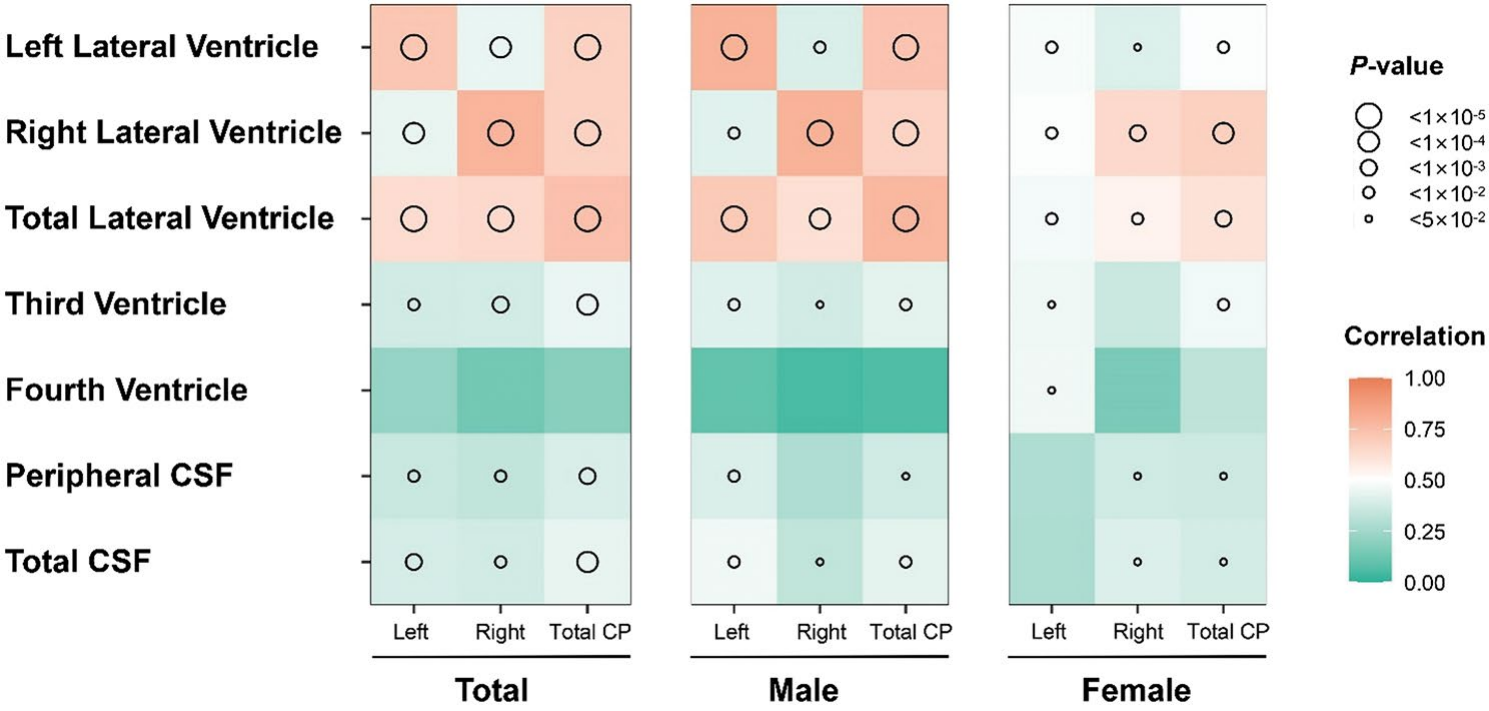
Heatmaps of the correlations between CP volume and brain ventricular system and CSF space metrics in different sex groups

The volumes of the left, right, and total lateral ventricles, third ventricle, peripheral CSF, and total CSF were significantly correlated with the corresponding CP volume (correlation coefficients r: 0.362 to 0.799, *p* < 0.05), with a *p* value of <0.01 for the relationship between the lateral ventricular system and CP. Moreover, the correlation between right CP and right lateral ventricle volume was generally stronger in males than in females (male/female r: 0.798 vs. 0.649, *p* < 1 × 10 ^− 4^) (Fig. 4).

### Estimation of regional gene expression and lasso regression analysis

**Fig. 5:**
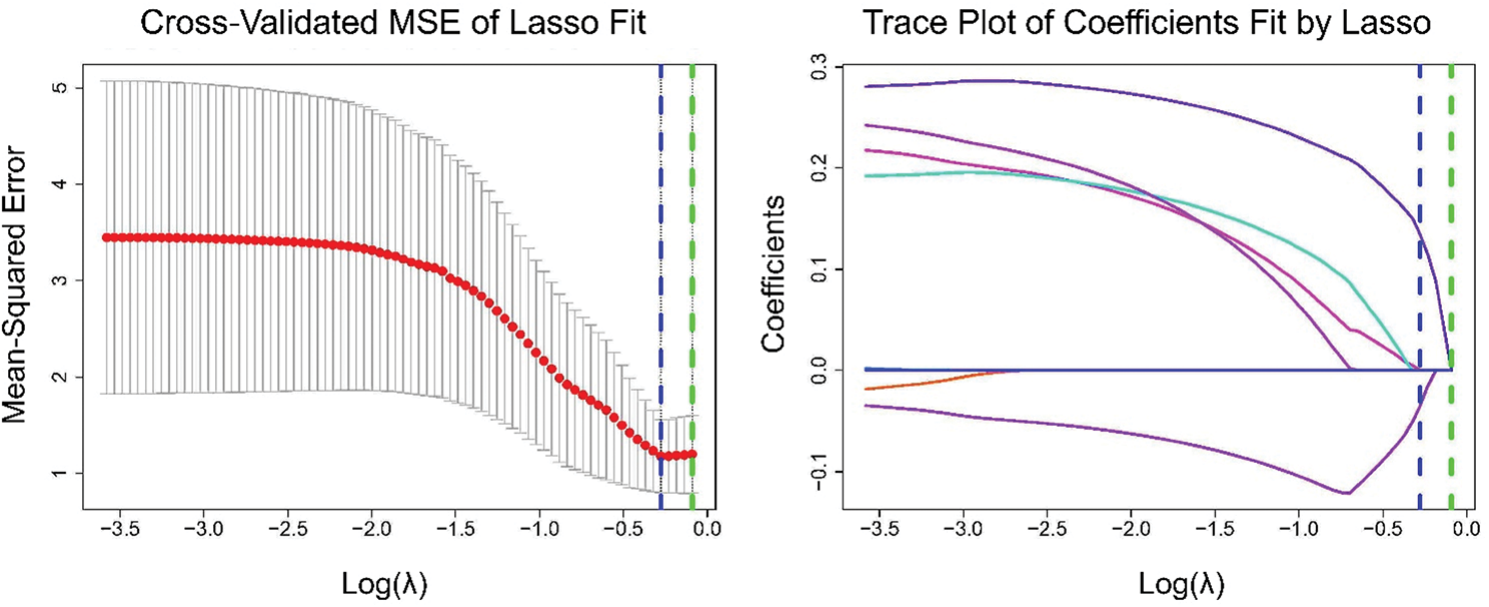
Cross-validated mean squared error (MSE) of the Lasso fit and the trace plot of coefficients selected by Lasso. The green line represents LambdaMinMSE, and the blue line represents Lambda1SE

To examine sex-related differences within the ventricular system, a gene expression matrix was constructed, incorporating transcriptional data from seven brain regions, including the left and right CPs, lateral ventricles, third and fourth ventricles, and peripheral CSF, encompassing a total of 15,633 genes. Two genes were identified as significantly influencing sex-related differences in volumes of the ventricular system (Fig. 5). The regression coefficients for these genes were calculated as follows: *DERL2* (0.1319) and *MRPL48* (-0.0370).

**Fig. 6:**
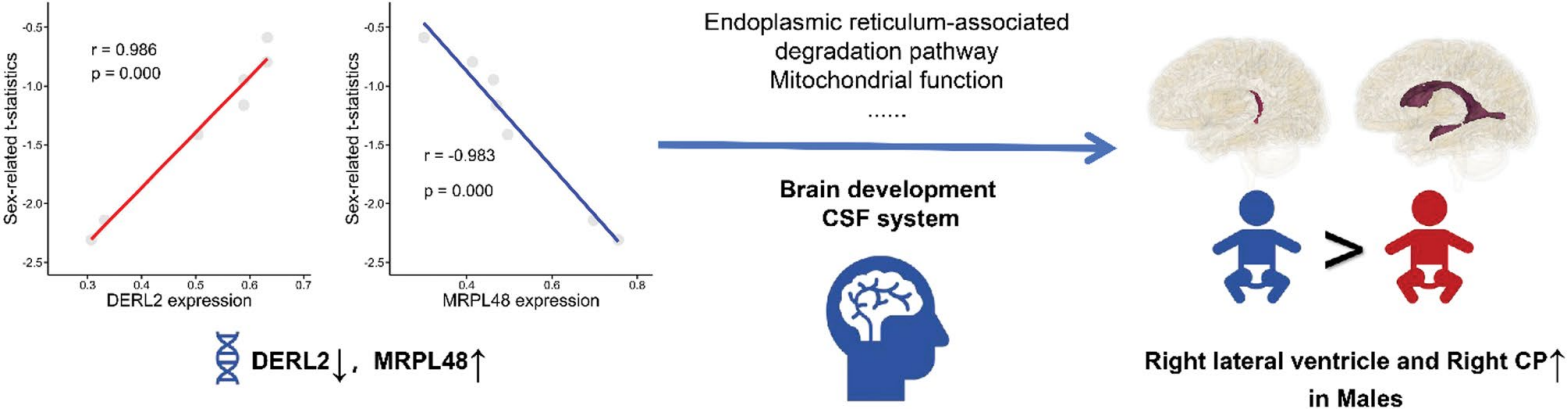
Overview of the results. Male neonates present larger volumes and faster growth of the right lateral ventricle, likely linked to corresponding CP volume and growth pattern. The differential expression of DERL2 and MRPL48 may contribute to these sex-related variations in the CSF system, suggesting a molecular basis for sex-specific brain development

We performed a linear correlation analysis (Fig. 6) between the expression levels of these genes and the sex-related t-statistics derived from our imaging data (with coding: female = 2, male = 1, and all t-values < 0). The analysis revealed a positive correlation between *DERL2* expression and the sex-related t-values, while *MRPL48* expression showed a negative correlation. These findings suggest that *DERL2* is relatively downregulated, and *MRPL48* relatively upregulated, in association with the sex-related pattern observed in CSF metrics.

## Discussion

This study of 75 full-term healthy neonates utilized deep-learning automated segmentation to analyze metrics of the brain ventricular system and CSF. It was found that males exhibit larger ventricular CSF spaces and faster growth rates than do females. Even after adjusting for TBV, males continued to demonstrate a positive linear growth trend, with females showing a relatively stable pattern. We hypothesize that these differences are partly due to variations in the volume and growth patterns of the CP between sexes.

### CP as a source of CSF differences

The CP, which is the primary source of CSF, is essential for the secretion and transport of CSF and for maintaining the blood–CSF barrier [28]. During embryo development, the CP first appears in the fourth ventricle, followed by the simultaneous formation of the CP in the lateral ventricles, and lastly in the third ventricle [5]. CP epithelial cells have been implicated in the pathophysiology of various neurological conditions, including neurodegenerative diseases, hydrocephalus, and stroke [29]. While MRI studies have predominantly focused on the role of the CP in neuroinflammation in conditions such as multiple sclerosis, where an increased CP volume is correlated with greater disability and cognitive decline [30], its growth trajectory and sex-related differences at the macrostructural level in neonates have been largely overlooked. The CP was segmented in our study primarily into the lateral ventricles due to the limited visibility and impact of that in third or fourth ventricles [31]. Our study suggests that sex-related differences in CP volume and growth patterns are a key factor contributing to the observed variations in the CSF system between male and female neonates. Similar sex-related differences were found both in right lateral ventricles and right CP volume. There was also a stronger correlation between right CP and right lateral ventricle volumes in males than in females. Moreover, the growth pattern of the CP closely aligns with those of the corresponding lateral ventricles, third ventricle, peripheral CSF, and the total CSF volume, indicating a possible mechanistic link. Previous research has shown that CP epithelial cells contribute to neurodevelopment and cognitive function [6]. which underscores the potential impact of CP differences on CSF dynamics and brain development.

### Larger volume and faster growth of lateral ventricles in males

Our study has confirmed that males have larger lateral ventricles with faster growth rates, especially for the right lateral ventricle. The lateral ventricles in fetuses were previously reported to be 22.1% larger in males [9]. A study focusing on white matter and subcortical gray matter, and which did not consider the CSF volume, found that 1-month-old infants underwent age-related changes, with males showing a larger TBV and region-specific differences after correcting for TBV [12]. Research involving full-term or term-equivalent preterm infants aged 2 to 90 days has identified lateral-ventricle asymmetry and a significant interaction between sex and age, although the CP was not examined [32]. Additionally, a study of infants aged 3–13 months found that males had larger lateral ventricles than females, with this sex-related difference disappearing when the volumes were normalized [33]. This is consistent with findings in studies of children aged 0–11 years [34] and 0–18 years [10]. While these studies have consistently shown that males have a larger ventricular system, this sex-related difference is somewhat diminished when TBV is accounted for. Our findings suggest that sex-related differences in the ventricular system and CSF also exist during the neonatal period, aligning with those observed at other ages and in other brain regions during development. While the left lateral ventricle is generally larger than the right lateral ventricle, our findings indicate that the key sex-related difference in the lateral ventricles is on the right side, which aligns with the difference in right CP volume. This suggests that factors beyond TBV, such as sex-related brain lateralization, contribute to these differences. Notably, significant brain asymmetries and sex-related differences have been observed early in childhood [35], with structures such as the globus pallidus and putamen that are part of the ventricular wall [36] and influence cognitive function.

Regarding development rates, previous studies have shown that the absolute volume of CSF increases significantly during the second half of the gestation period, while the volume ratio of CSF to brain parenchyma stabilizes over time [9]. The ventricle volume was reported to increase by 350% from a GA of 15 weeks to birth, and by 86% from birth to 1.25 years, while it decreased by 18% from 1.25 to 3.5 years [34]. CSF volume hasn’t reached the peak during the pediatric age range [10]. Our findings, consistent with the stabilizing yet increasing growth of CSF space from gestation to birth, have revealed the significant sex-related differences in the early postnatal growth patterns of the lateral ventricles after adjusting for GA. This information might be particularly useful when developing optimal protocols for monitoring sex-related development during the neonatal period.

However, it is crucial to interpret these findings with caution. In the cohort, the majority of MRI scans were acquired within the first week after birth, resulting in a highly skewed distribution of postnatal age. Although significant associations were detected between postnatal age and CSF-related metrics, these likely reflect developmental dynamics specific to the immediate postnatal period, rather than the full neonatal stage. Therefore, our results should be viewed as preliminary and time-specific. Future research incorporating more temporally distributed data across the neonatal period—and ideally longitudinal designs—will be essential to better characterize the evolving trajectory of CSF space development and its sex-specific patterns.

### No difference in total CSF volume with relatively faster growth of peripheral CSF in males

Our study suggests that peripheral CSF constitutes a substantial portion of total CSF, likely mitigating sex-related differences in lateral ventricles, leading to the absence of differences in total CSF volume. The growth pattern of peripheral CSF aligns with the sex-related differences observed for other CSF space metrics: while males continue to show growth, the values for females stabilize. Our findings reveal significant sex-related differences in CSF distribution patterns and growth rates. These differences may be influenced by biochemical factors such as the osmolarity, pressure, and flow rate of CSF [6]. Previous research studies have found that males generally have higher CSF protein content and albumin quotient values across various pathologies and age groups, and are more likely to experience blood–CSF barrier dysfunction [2].

### Other findings in third and fourth ventricles, and in EI

The third ventricle showed significant growth in both its absolute volume and its percentage relative to TBV. Previous studies indicate that the GA at birth does not significantly influence the size of the third ventricle during the first 3 months of life [32]. However, a study involving 700 children found that during normal development the third ventricle exhibited a segmental increase from birth to the age of 18 years, with no influence of sex [37]. Our findings suggest that the third ventricle exhibits a delayed growth phase during fetal development, followed by rapid expansion during the neonatal period. Positioned between the two halves of the thalamus, the third ventricle is essential for CSF circulation [38] and participates in the contributions of the thalamus to the regulation of vital functions [38, 39] and to the development of higher brain functions [40].

In contrast, the percentage of the fourth ventricle relative to TBV exhibited a declining trend in the present study. Since the fourth ventricle serves as a critical junction between the ventricular system and the subarachnoid space, its obstruction can lead to rapid CSF accumulation that represents a medical emergency [41]. The early detection of subtle changes in the fourth ventricle may therefore have important clinical implications for the prompt diagnosis and management of neurodevelopmental disorders.

The EI is commonly used in clinical practice as a rapid alternative to MRI volumetric quantification for conditions such as pediatric hydrocephalus [42], normal-pressure hydrocephalus [43], and Alzheimer’s disease (AD) [44]. The present study has revealed significant sex-related differences in the EI, suggesting that including sex-related differences when calculating the EI will improve clinical diagnoses.

This study was subject to several limitations that should be addressed in future research. First, the smallness of the sample reduces the generalizability of our findings. Increasing the sample size in future studies will enhance the robustness of the results and allow for more-nuanced analyses of sex-related differences in the ventricular system and CSF development. Second, the autosegmentation of small and intricate structures such as the third ventricle, fourth ventricle, and CP may not be as precise as metrics involving structures such as white matter, which potentially obscures subtle differences. Employing more-advanced imaging techniques or manual segmentation could improve accuracy. Third, our study did not account for socioeconomic status or maternal health during pregnancy, and these factors could influence neonatal brain development. Future research should include matched cohorts to control for these variables. Finally, this was a single-center study, which may reduce the external validity of our findings. Multicenter longitudinal studies are needed to confirm these results and further explore the sex-related differences in CSF space metrics.

### Insights from gene expression

Using Lasso regression to predict sex-related differences in the left and right CPs, lateral ventricles, third and fourth ventricles, and peripheral ventricles based on regional gene expression, we identified two relatively important genes: *DERL2* and *MRPL48*.

DERL2 is a key component of the endoplasmic reticulum quality control system and plays a essential role in the degradation of misfolded glycoproteins through the ER-associated degradation (ERAD) pathway [45, 46]. Maintaining ER homeostasis is critical for normal brain development and memory formation [47], whereas disruption of this balance has been implicated in neurodegenerative diseases characterized by the accumulation of misfolded proteins [47]. In AD model mice, physical exercise has been shown to upregulate *DERL2* expression in the hippocampus [48]. Global knockout of *DERL2* results in perinatal lethality, and the few surviving mice exhibit skeletal dysplasia due to defective collagen secretion in costal chondrocytes [49]. Conditional knockout of *DERL2* in the CNS impairs postnatal brain development, particularly affecting the cerebellum and striatum, and leads to motor deficits [50]. *DERL2* deficiencies has also been shown to inhibit neurite outgrowth and suppress sterol regulatory element-binding protein 2-mediated brain cholesterol biosynthesis—an essential process for glial proliferation, neurite extension, and microtubule stability [51]. In parallel, CSF system also contributes significantly to the clearance of misfolded proteins. Impaired meningeal lymphatic drainage, especially during aging or in AD, can exacerbate toxic protein accumulation in CNS [52].

MRPL48 is a component of the 39 S large subunit of the mammalian mitochondrial ribosome and plays a critical role in mitochondrial translation [53]. Encoded by nuclear DNA, *MRPL48* exhibits region-specific expression in the brain, with significantly higher levels observed in the frontal cortex compared to the hippocampus or hypothalamus in rats [54]. In addition, *MRPL48* has been implicated in the development of nervous and endocrine system, with its dysregulation linked to hereditary, metabolic, and neurological disorders [55]. In forebrain mineralocorticoid receptor knockout mice, *MRPL48* expression is downregulated, while glucocorticoid receptor expression is slightly upregulated [56]. Notably, *MRPL48* displays a female-specific paternal expression bias in the medial preoptic area [57] and is differentially expressed in patients with schizophrenia compared to healthy controls [58]. Moreover, a study using cDNA microarray and proteomic analyses in rats have shown that gene expression in the choroid plexus is enriched in pathways related to mitochondrial dysfunction and oxidative phosphorylation [59].

In conclusion, the downregulation of *DERL2* and upregulation of *MRPL48* observed in our study may contribute to the altered clearance of misfolded proteins and altered energy metabolism.These molecular alterations could ultimately underlie sex-related morphological differences in the ventricular system (Fig. 6).

## Conclusion

This study has revealed sex-related differences in the development of the brain ventricular system and CSF in neonates. Right lateral ventricles and right CP were larger in males, with a stronger correlation between their volumes compared to females during early postnatal age. No difference was found in total CSF volume, with relatively faster growth of peripheral CSF and CP in males. The observed sex-related differences in CP volume and significantly correlated growth patterns suggest that the CP plays key roles in driving these differences. Additionally, the downregulation of *DERL2* and upregulation of *MRPL48* may contribute to these sex-specific variations in the CSF system, highlighting a potential molecular basis for the observed differences in brain development.

These findings contribute to our understanding of neonatal brain development and underscore the importance of considering sex as an influencing factor in future research. The observed morphological differences may represent some of the earliest structural manifestations of sex-based brain differentiation, which could help in identifying atypical developmental trajectories at an early stage. Moreover, the associated gene expression patterns provide insights into the molecular mechanisms underlying sex differences in brain development and may help explain sex-specific vulnerabilities to neurodevelopmental disorders. Further study involving larger and more diverse cohorts is warranted to validate and generalize the present findings. In addition, we plan to incorporate individual-level transcriptomic data to confirm our gene expression observations. Furthermore, we will collect both short- and long-term neurobehavioral developmental assessments to explore their potential correlations with the observed structural and genetic differences.

## Electronic supplementary material

Below is the link to the electronic supplementary material.

Supplementary Material 1

## Data Availability

No datasets were generated or analysed during the current study.
