# Supplementary material for: Sex-related differences and associated transcriptional signatures in the brain ventricular system and cerebrospinal fluid development in full-term neonates
Source: Biol Sex Differ. 2025 May 25;16:35. doi: 10.1186/s13293-025-00719-2 (PMC12103790; doi:10.1186/s13293-025-00719-2)
Supplement: Supplementary file 1 — Supplementary Material 1 [file 13293_2025_719_MOESM1_ESM.docx]

**Supplementary materials**

**1. Segmentation workflow and validation of 3D T1-weighted MRI using the uAI Research Portal (uRP)**

In our study, we processed three-dimensional T1-weighted images using the United Imaging Intelligence Research Portal (uAI Research Portal, uRP). United Imaging, the leading MRI provider in China since 2020, has consistently provided advanced technologies. The uRP platform, which has supported over 30 academic publications, integrates the VB-Net segmentation toolkit, capable of processing high-resolution MR images for multi-level brain structure segmentation. The workflow includes: (1) bias field correction, (2) skull stripping, (3) tissue segmentation (white matter, gray matter, CSF), (4) bilateral segmentation, and (5) segmentation of 109 sub-regions across multiple brain areas, including the temporal, frontal, parietal, occipital lobes, cerebellum, subcortical gray matter, and ventricles. We added visual representations of segmentation results to clearly demonstrate the accuracy and reliability of the uRP-based segmentation.


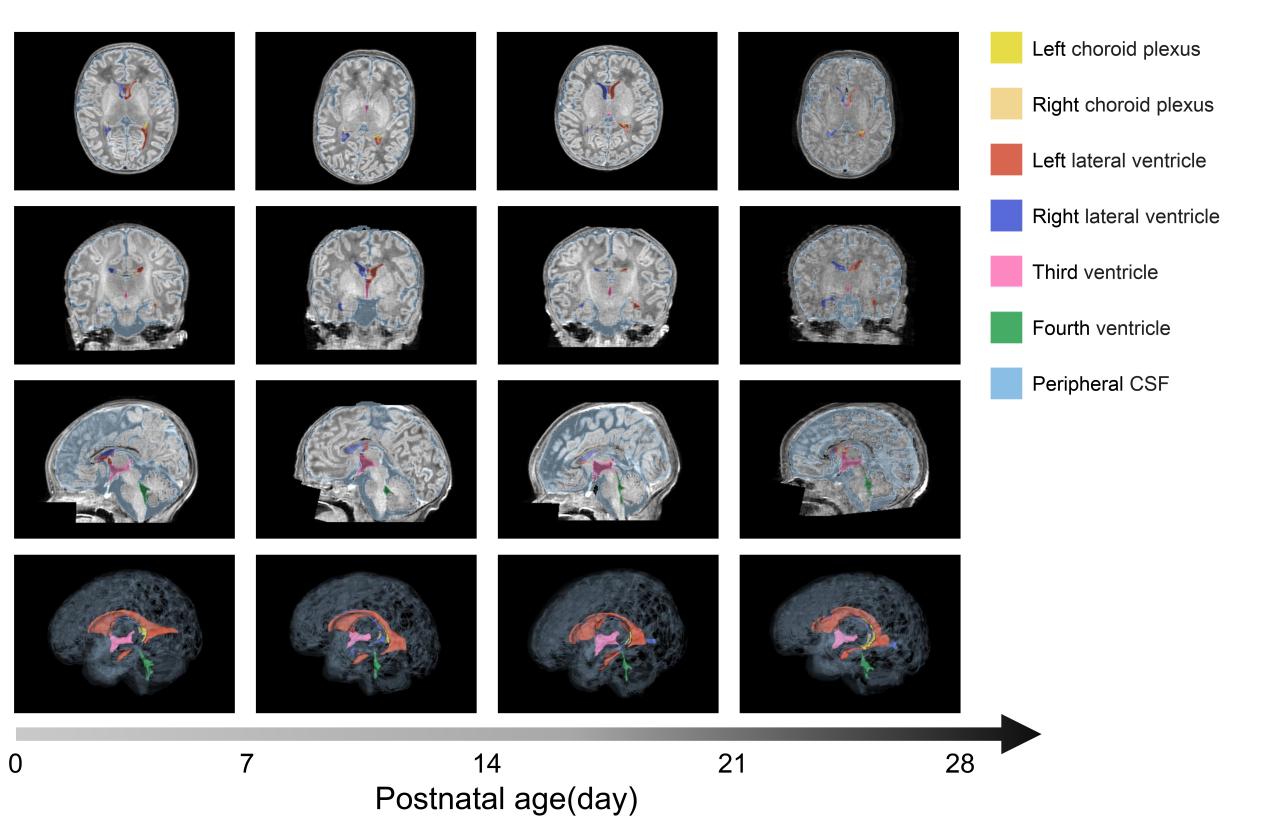


**Supplementary Figure 1** Image segmentation results of newborns at different age intervals (0-7 days, 7-14 days, 14-21 days, and 21-28 days) using uRP. The figure presents coronal, sagittal, and axial views, alongside 3D reconstructions. Segmentation outcomes include the left and right choroid plexus, left and right lateral ventricles, third ventricle, fourth ventricle, and peripheral cerebrospinal fluid.

1. **Detailed information of statistical methods & results applied to each ventricular system volumes and CSF spaces.**

**Supplementary Table 1 Specific statistical methods Applied to each brain ventricular system and CSF metrics**

| Brain ventricular system and CSF metrics | male | | female | | **Test type** | *p* |
| --- | --- | --- | --- | --- | --- | --- |
|  | mean | standard deviation | mean | standard deviation |  |  |
| PNA | 5.5682 | 8.0735 | 5.0323 | 6.8871 | Mann-Whitney U | 0.5074 |
| EI | 0.2514 | 0.0232 | 0.2409 | 0.0204 | t-test (equal variances) | 0.0477 |
| Left CP (cm^3^) | 0.1362 | 0.0701 | 0.1233 | 0.0476 | Mann-Whitney U | 0.4776 |
| Right CP (cm^3^) | 0.1610 | 0.0662 | 0.1313 | 0.0388 | Mann-Whitney U | 0.0378 |
| Total CP (cm^3^) | 0.2972 | 0.1203 | 0.2546 | 0.0720 | Mann-Whitney U | 0.1113 |
| Left Lateral Ventricle (cm^3^) | 2.5558 | 0.8655 | 2.3374 | 0.5968 | Mann-Whitney U | 0.2748 |
| Right Lateral Ventricle (cm^3^) | 2.4168 | 0.9763 | 2.0379 | 0.4469 | Mann-Whitney U | 0.0339 |
| Total Lateral Ventricle (cm^3^) | 4.9726 | 1.6569 | 4.3753 | 0.9757 | Mann-Whitney U | 0.0368 |
| Third Ventricle (cm^3^) | 0.3020 | 0.1020 | 0.2822 | 0.0734 | Mann-Whitney U | 0.3356 |
| Fourth Ventricle (cm^3^) | 0.4185 | 0.0962 | 0.3888 | 0.0778 | t-test (equal variances) | 0.1591 |
| Peripheral CSF (cm^3^ | 71.9131 | 14.5709 | 70.0039 | 13.0776 | t-test (equal variances) | 0.5620 |
| CSF (cm^3^) | 77.6063 | 15.3850 | 75.0502 | 13.6173 | t-test (equal variances) | 0.4603 |
| TBV | 358.141 | 39.511 | 336.650 | 36.299 | Mann-Whitney U | 0.0026 |
| Left ChP/TBV (%) | 0.0379 | 0.0192 | 0.0366 | 0.0132 | Mann-Whitney U | 0.8265 |
| Right ChP/TBV (%) | 0.0446 | 0.0161 | 0.0391 | 0.0108 | Welch's t-test | 0.0799 |
| ChP/TBV (%) | 0.0826 | 0.0301 | 0.0756 | 0.0192 | Mann-Whitney U | 0.3270 |
| Left Lateral Ventricle/TBV (%) | 0.7168 | 0.2523 | 0.6934 | 0.1439 | Mann-Whitney U | 0.8265 |
| Right Lateral Ventricle/TBV (%) | 0.6716 | 0.2513 | 0.6065 | 0.1151 | Mann-Whitney U | 0.3946 |
| Total Lateral Ventricle/TBV (%) | 1.3884 | 0.4404 | 1.2999 | 0.2346 | Mann-Whitney U | 0.5817 |
| Third Ventricle/TBV (%) | 0.0839 | 0.0256 | 0.0831 | 0.0152 | Welch's t-test | 0.8653 |
| Fourth Ventricle/TBV (%) | 0.1180 | 0.0288 | 0.1162 | 0.0241 | t-test (equal variances) | 0.7706 |
| Peripheral CSF/TBV (%) | 20.0488 | 3.2347 | 20.7479 | 2.9538 | Mann-Whitney U | 0.5243 |

**Supplementary Table 2 Correlation between brain CSF system and postnatal age in different sex groups**

| Brain ventricular system and CSF metrics | Male | | | | Female | | | |
| --- | --- | --- | --- | --- | --- | --- | --- | --- |
|  |  |  | GA adjusted | |  |  | GA adjusted | |
|  | r | *p* value | **r** | ***p* value** | r | *p* value | **r** | ***p* value** |
| EI | 0.177 | 0.251 | 0.169 | 0.273 | 0.314 | 0.086 | 0.318 | 0.081 |
| Left CP (cm^3^) | 0.321 | 0.034* | 0.321 | 0.034* | 0.325 | 0.074 | 0.319 | 0.080 |
| Right CP (cm^3^) | 0.301 | 0.047* | 0.283 | 0.063 | 0.465 | 0.009* | 0.468 | 0.008* |
| Total CP (cm^3^) | 0.348 | 0.021* | 0.334 | 0.027* | 0.449 | 0.011* | 0.454 | 0.010* |
| Left Lateral Ventricle (cm^3^) | 0.330 | 0.029* | 0.358 | 0.017* | 0.356 | 0.049* | 0.374 | 0.038* |
| Right Lateral Ventricle (cm^3^) | 0.465 | 0.002* | 0.468 | 0.001* | 0.427 | 0.017* | 0.463 | 0.009* |
| Total Lateral Ventricle (cm^3^) | 0.434 | 0.003* | 0.488 | 0.001* | 0.432 | 0.015* | 0.457 | 0.010* |
| Third Ventricle (cm^3^) | 0.622 | 0.000* | 0.621 | 0.000* | 0.624 | 0.000* | 0.588 | 0.001* |
| Fourth Ventricle (cm^3^) | 0.208 | 0.175 | 0.215 | 0.161 | 0.071 | 0.704 | 0.073 | 0.695 |
| Peripheral CSF (cm^3^ | 0.549 | 0.000* | 0.604 | 0.000* | 0.729 | 0.000* | 0.736 | 0.000* |
| CSF (cm^3^) | 0.586 | 0.000* | 0.619 | 0.000* | 0.726 | 0.000* | 0.757 | 0.000* |
| Left CP/TBV (%) | 0.260 | 0.089 | 0.220 | 0.152 | 0.120 | 0.521 | 0.128 | 0.494 |
| Right CP/TBV (%) | 0.167 | 0.277 | 0.150 | 0.333 | 0.158 | 0.396 | 0.176 | 0.344 |
| CP/TBV (%) | 0.232 | 0.129 | 0.265 | 0.083 | 0.122 | 0.514 | 0.195 | 0.293 |
| Left Lateral Ventricle/TBV (%) | 0.175 | 0.256 | 0.149 | 0.333 | 0.123 | 0.511 | 0.120 | 0.521 |
| Right Lateral Ventricle/TBV (%) | 0.270 | 0.076 | 0.279 | 0.067 | 0.162 | 0.384 | 0.217 | 0.241 |
| Total Lateral Ventricle/TBV (%) | 0.213 | 0.164 | 0.236 | 0.123 | 0.190 | 0.307 | 0.219 | 0.237 |
| Third Ventricle/TBV (%) | 0.502 | 0.001* | 0.501 | 0.001* | 0.449 | 0.011* | 0.469 | 0.008* |
| Fourth Ventricle/TBV (%) | 0.024 | 0.877 | 0.015 | 0.925 | -0.265 | 0.149 | -0.236 | 0.201 |
| Peripheral CSF/TBV (%) | 0.404 | 0.007* | 0.414 | 0.005* | 0.466 | 0.008* | 0.500 | 0.004* |
| CSF/TBV (%) | 0.441 | 0.003* | 0.443 | 0.003* | 0.528 | 0.002* | 0.508 | 0.004* |

Adjusted method: residuals after linear regression with GA

Abbreviations: EI, Evans Index; CP, choroid plexuses; CSF, cerebrospinal fluid; TBV, total brain volume

r denotes the correlation coefficient in the linear relationship

* p<0.05

1. **Handling of outliers and their impact on sex-related differences of brain ventricular system metrics**

Upon re-examining the data, we confirmed the presence of outliers in both the male and female groups. As shown in Figure 2, the blue circles indicate outliers identified using MATLAB’s default method based on the 1.5 * IQR (Interquartile Range) rule: any data point above Q3 + 1.5 * IQR or below Q1 - 1.5 * IQR is considered an outlier. These outliers, marked with blue circles, significantly deviate from the rest of the data and may represent exceptional observations.

For the right lateral ventricle, three outliers were identified in the male group and one in the female group. After excluding these outliers, significant sex-related differences in the right choroid plexus and right lateral ventricle volumes persisted (see Table 3).

For the left lateral ventricle, three outliers were found in the male group and three in the female group. After excluding these outliers, significant differences in the right choroid plexus and right lateral ventricle volumes remained, while the difference in the left lateral ventricle remained non-significant (see Table 4).

**Supplementary Table 3 Sex-related differences in metrics of the brain ventricular system and cerebrospinal fluid (CSF) after excluding outliers in the right lateral ventricle**

| Brain ventricular system and CSF metrics | female | | male | | test_type | *p* |
| --- | --- | --- | --- | --- | --- | --- |
|  | mean | sd | mean | sd |  |  |
| PNA | 4.567 | 6.490 | 4.780 | 7.528 | Mann-Whitney U | 0.325 |
| EI | 0.239 | 0.019 | 0.248 | 0.021 | t-test (equal variances) | 0.069 |
| Left CP | 0.121 | 0.047 | 0.127 | 0.059 | Mann-Whitney U | 0.633 |
| Right CP | 0.128 | 0.035 | 0.152 | 0.053 | Welch's t-test | 0.025* |
| Total CP | 0.249 | 0.066 | 0.279 | 0.096 | Mann-Whitney U | 0.162 |
| Left Lateral Ventricle | 2.293 | 0.554 | 2.405 | 0.625 | Mann-Whitney U | 0.373 |
| Right Lateral Ventricle | 1.997 | 0.390 | 2.211 | 0.565 | Mann-Whitney U | 0.048* |
| Total Lateral Ventricle | 4.290 | 0.867 | 4.615 | 1.003 | Mann-Whitney U | 0.051 |
| Third Ventricle | 0.281 | 0.074 | 0.293 | 0.099 | Mann-Whitney U | 0.526 |
| Fourth Ventricle | 0.387 | 0.079 | 0.418 | 0.100 | t-test (equal variances) | 0.169 |
| Peripheral CSF | 69.707 | 13.195 | 71.230 | 14.631 | t-test (equal variances) | 0.653 |
| CSF | 74.665 | 13.677 | 76.556 | 15.164 | t-test (equal variances) | 0.591 |
| Left ChP-TBV percent | 0.036 | 0.013 | 0.036 | 0.016 | Mann-Whitney U | 0.977 |
| Right ChP-TBV percent | 0.038 | 0.010 | 0.042 | 0.014 | t-test (equal variances) | 0.188 |
| ChP-TBV percent | 0.075 | 0.019 | 0.078 | 0.025 | Mann-Whitney U | 0.476 |
| Left Lateral Ventricle-TBV percent | 0.685 | 0.139 | 0.677 | 0.188 | Mann-Whitney U | 0.583 |
| Right Lateral Ventricle-TBV percent | 0.599 | 0.108 | 0.618 | 0.142 | t-test (equal variances) | 0.524 |
| Total Lateral Ventricle-TBV percent | 1.284 | 0.220 | 1.296 | 0.267 | t-test (equal variances) | 0.840 |
| Third Ventricle-TBV percent | 0.083 | 0.015 | 0.081 | 0.024 | Welch's t-test | 0.731 |
| Fourth Ventricle-TBV percent | 0.116 | 0.025 | 0.118 | 0.029 | t-test (equal variances) | 0.784 |
| Peripheral CSF-TBV percent | 20.761 | 3.003 | 19.931 | 3.289 | Mann-Whitney U | 0.414 |
| CSF-TBV percent | 22.244 | 3.067 | 21.427 | 3.371 | Mann-Whitney U | 0.506 |

* p<0.05

**Supplementary Table 4 Sex-related differences in metrics of the brain ventricular system and cerebrospinal fluid (CSF) after excluding outliers in the left lateral ventricle**

| Brain ventricular system and CSF metrics | female | | male | | test_type | *p* |
| --- | --- | --- | --- | --- | --- | --- |
|  | mean | sd | mean | sd |  |  |
| PNA | 4.357 | 6.561 | 5.146 | 7.751 | Mann-Whitney U | 0.566 |
| EI | 0.238 | 0.018 | 0.250 | 0.023 | t-test (equal variances) | 0.028* |
| Left CP | 0.113 | 0.036 | 0.125 | 0.058 | Welch's t-test | 0.271 |
| Right CP | 0.126 | 0.034 | 0.156 | 0.055 | Welch's t-test | 0.007* |
| Total CP | 0.239 | 0.054 | 0.281 | 0.097 | Mann-Whitney U | 0.057 |
| Left Lateral Ventricle | 2.187 | 0.361 | 2.378 | 0.561 | Welch's t-test | 0.090 |
| Right Lateral Ventricle | 1.963 | 0.366 | 2.320 | 0.836 | Mann-Whitney U | 0.014* |
| Total Lateral Ventricle | 4.150 | 0.659 | 4.699 | 1.239 | Mann-Whitney U | 0.016* |
| Third Ventricle | 0.274 | 0.072 | 0.296 | 0.102 | Mann-Whitney U | 0.325 |
| Fourth Ventricle | 0.387 | 0.082 | 0.418 | 0.100 | t-test (equal variances) | 0.180 |
| Peripheral CSF | 68.556 | 12.884 | 71.860 | 14.545 | t-test (equal variances) | 0.336 |
| CSF | 73.367 | 13.213 | 77.273 | 15.225 | Mann-Whitney U | 0.216 |
| Left ChP-TBV percent | 0.034 | 0.012 | 0.035 | 0.015 | Mann-Whitney U | 0.757 |
| Right ChP-TBV percent | 0.038 | 0.011 | 0.043 | 0.015 | t-test (equal variances) | 0.109 |
| ChP-TBV percent | 0.072 | 0.017 | 0.078 | 0.026 | Mann-Whitney U | 0.260 |
| Left Lateral Ventricle-TBV percent | 0.663 | 0.112 | 0.665 | 0.148 | t-test (equal variances) | 0.952 |
| Right Lateral Ventricle-TBV percent | 0.595 | 0.110 | 0.647 | 0.228 | Mann-Whitney U | 0.346 |
| Total Lateral Ventricle-TBV percent | 1.257 | 0.200 | 1.312 | 0.327 | Mann-Whitney U | 0.485 |
| Third Ventricle-TBV percent | 0.082 | 0.015 | 0.082 | 0.025 | Welch's t-test | 0.967 |
| Fourth Ventricle-TBV percent | 0.117 | 0.025 | 0.118 | 0.029 | t-test (equal variances) | 0.949 |
| Peripheral CSF-TBV percent | 20.603 | 2.981 | 20.042 | 3.330 | Mann-Whitney U | 0.693 |
| CSF-TBV percent | 22.059 | 3.033 | 21.554 | 3.459 | Mann-Whitney U | 0.813 |

* p<0.05


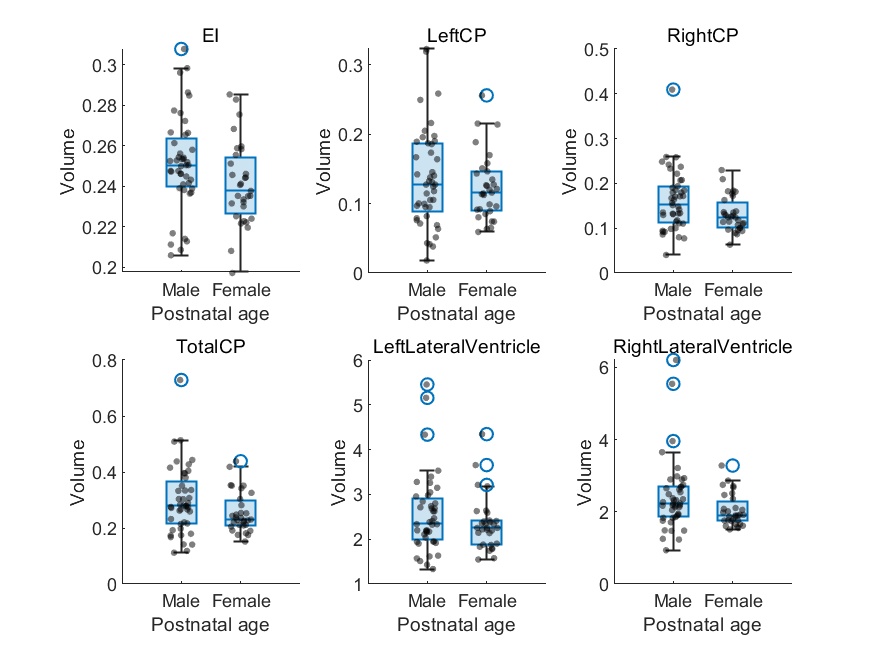


**Supplementary Figure2. Analysis of outliers**

Blue Circles represent MATLAB's default identified outliers. MATLAB uses the rule of 1.5 * IQR (Interquartile Range) to identify outliers: any point above Q3 + 1.5 * IQR or below Q1 - 1.5 * IQR is considered an outlier. In this plot, the blue circles mark these outliers, indicating that they deviate significantly from the rest of the data and may represent unusual or exceptional observations.

1. **Correction for Total CSF Volume**

we have conducted the analysis with an additional correction for total CSF volume. Our findings show that, after adjusting for total CSF volume, the differences that were previously significant become non-significant, which mirrors the results obtained after correcting for total brain volume (TBV).

**Supplementary Table 5 Sex-related differences in metrics of the brain ventricular system and cerebrospinal fluid (CSF) relative to CSF**

| Brain ventricular system and CSF metrics | male | | female | | **Test type** | *p* |
| --- | --- | --- | --- | --- | --- | --- |
|  | mean | standard deviation | mean | standard deviation |  |  |
| Left ChP/CSF (%) | 0.1741 | 0.0828 | 0.1657 | 0.0584 | Welch's t-test | 0.6085 |
| Right ChP/CSF (%) | 0.2087 | 0.0779 | 0.1773 | 0.0513 | Mann-Whitney U | 0.1034 |
| ChP/CSF (%) | 0.3827 | 0.1332 | 0.3430 | 0.0897 | Mann-Whitney U | 0.1780 |
| Left Lateral Ventricle/CSF (%) | 3.3323 | 1.0547 | 3.1452 | 0.6676 | Mann-Whitney U | 0.7687 |
| Right Lateral Ventricle/CSF (%) | 3.1165 | 1.0222 | 2.7544 | 0.5549 | Mann-Whitney U | 0.2194 |
| Total Lateral Ventricle/CSF (%) | 6.4488 | 1.7773 | 5.8996 | 1.1179 | Mann-Whitney U | 0.3486 |
| Third Ventricle/CSF (%) | 0.3838 | 0.0928 | 0.3778 | 0.0736 | t-test (equal variances) | 0.7631 |
| Fourth Ventricle/CSF (%) | 0.5546 | 0.1479 | 0.5296 | 0.1259 | t-test (equal variances) | 0.4465 |
| Peripheral CSF/CSF (%) | 92.6128 | 1.8078 | 93.1931 | 1.1840 | Mann-Whitney U | 0.3062 |

**
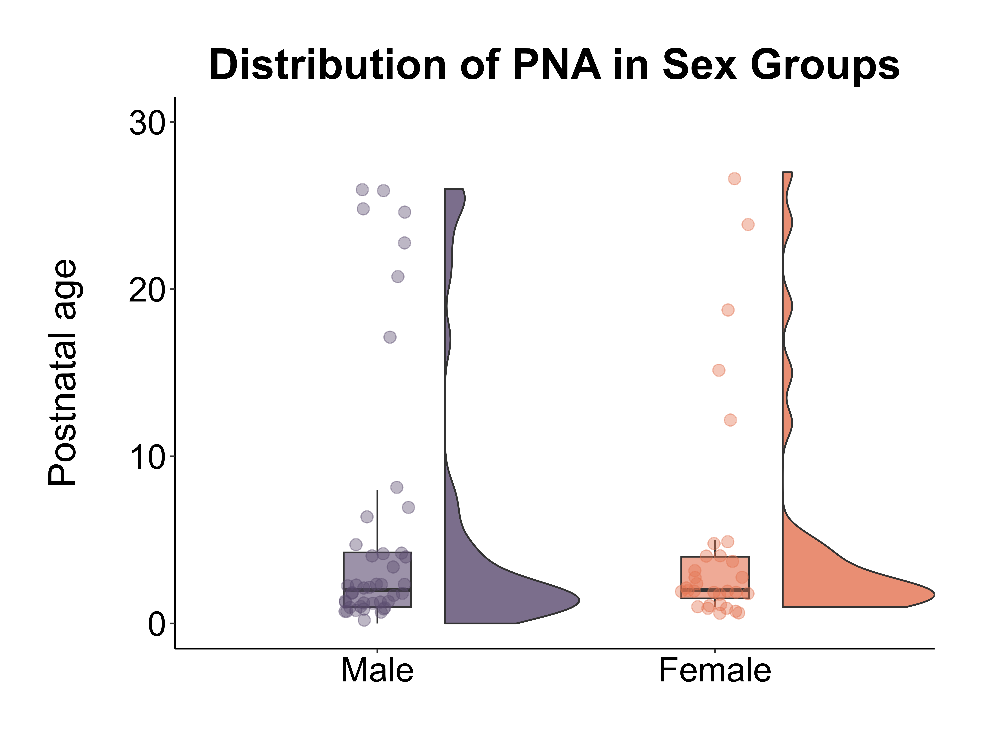
Supplementary Figure3. The distribution of PNA in different sex groups**
